# Heterogeneous Fusion of Camera and mmWave Radar Sensor of Optimizing Convolutional Neural Networks for Parking Meter System

**DOI:** 10.3390/s23084159

**Published:** 2023-04-21

**Authors:** Chi-Chia Sun, Yong-Ye Lin, Wei-Jia Hong, Gene-Eu Jan

**Affiliations:** 1 Department of Electrical Engineering, National Formosa University, Huwei 632, Taiwan; 2 Smart Machine and Intelligent Manufacturing Research Center, National Formosa University, Huwei 632, Taiwan; 3 Department of Electro-Optical Engineering, National Formosa University, Huwei 632, Taiwan; 4 Department of Electrical Engineering, National Taipei University, New Taipei City 237, Taiwan

**Keywords:** parking meter, embedded computer, convolutional neural networks, mmWave radar

## Abstract

In this article, a novel heterogeneous fusion of convolutional neural networks that combined an RGB camera and an active mmWave radar sensor for the smart parking meter is proposed. In general, the parking fee collector on the street outdoor surroundings by traffic flows, shadows, and reflections makes it an exceedingly tough task to identify street parking regions. The proposed heterogeneous fusion convolutional neural networks combine an active radar sensor and image input with specific geometric area, allowing them to detect the parking region against different tough conditions such as rain, fog, dust, snow, glare, and traffic flow. They use convolutional neural networks to acquire output results along with the individual training and fusion of RGB camera and mmWave radar data. To achieve real-time performance, the proposed algorithm has been implemented on a GPU-accelerated embedded platform Jetson Nano with a heterogeneous hardware acceleration methodology. The experimental results exhibit that the accuracy of the heterogeneous fusion method can reach up to 99.33% on average.

## 1. Introduction

In an era of science and technology, flourishing methodology has become an emerging field of research in the 21st century. Many hot topics such as artificial intelligence, robotics, cloud computing, and big data have already produced a revolution in the field of flourishing methodology. Furthermore, it will continue to change our lives. For example, machine learning (ML) provides a computer algorithm that can be enhanced based on experience and is a subgroup of artificial intelligence. In order to improve automatically through experience, ML algorithms construct a mathematical model based on training data that would make predictions or decisions without being supervised [1,2]. Lately, in the past few years, machine learning and artificial intelligence have manifested their great utility and effectiveness in fathoming many real-time computationally-concerted problems. For instance, in image processing, to understand the position and type of image, the detection and classification topology frequently uses CIFAR10, CIFAR100, and ImageNet et al. [3,4]. An example of region segmentation is the significant interest in designing the indoor robot-like unmanned ground vehicle [5]. In the robotic control system, the design of inflated performance ground range detection calculation plays a very important role. The advanced driver assistance system (ADAS) [6] is another major application of region segmentation in real-time inference. The ADAS is the methodology of image segmentation that assists a driver or human-less driving system, which ensures the following of the driving rules and provides a warning about any kind of obstacle to avoid the probability of vehicle accidents. Nowadays, the machine learning algorithm has an ample range of applications, where that is tough or impractical to evolve conventional algorithms to perform required jobs such as email filtering and computer vision.

In recent years, unmanned roadside parking toll systems and other related payment technology applications have begun to combine these technologies with different types of field applications [7,8]. However, the biggest problem encountered is the poor efficiency of image recognition and radar detection. For example, the current trial operation of unmanned roadside parking toll systems is often caused by radar detection errors. If the charging information is wrong or the camera is blurry, it is difficult to overcome the problem of different scenarios without using machine-learning-recognition technology.

Side by side with customary machine learning algorithms, deep learning has been widely and successfully applied in different fields, such as image recognition [9,10,11], speech recognition [12], and face recognition [13,14,15]. Convolutional neural network (CNN) architecture is very often used for deep learning. CNN architecture has has excellent image recognition. In 1989, the first convolutional neural network architecture was proposed by LeCun et al. [16], which is known as LeNet-5. The LeNet-5 is mainly used for handwriting recognition in text-edited data. However, due to issues such as parameters size, gradients, and the insufficiency of hardware equipment, the costs and benefits are not consistent. Deep learning was not popular with users at the time. Important developments occurred prior to 2012. Krizhevsky et al. proposed a new convolutional neural network architecture called AlexNet [17] and introduced dropout [18] to overcome the issue of overfitting in the convolutional neural network. Numerous researchers have also suggested some new deep convolutional neural network architectures. Over time, the neural network model has grown, and the number of network layers continues to increase, such as from AlexNet with 8 layers to VGG with 16 layers, from VGG with 16 layers [19] to GoogLeNet [20] with 22 layers, from 22 layers to 152 layers of ResNet [21], and more. There are thousands of layers of ResNet and DenseNet [22]. Although the overall neural network model improves performance, it also increases the efficiency of the network model. In 2017, the Google team proposed MobileNet [23]. The MobileNet network architecture mainly uses Depthwise Separable Convolution instead of the traditional convolution method. This method can effectively reduce the size of the model without causing a misjudgment rate.

The mmWave millimeter-wave radar is a sensing technology that provides information about features such as range, reflection angle, and speed with the direction of detecting objects. It is a non-contact technology that works in the 30–300 GHz spectrum range since the wavelength of this technology is small. Therefore, it can easily penetrate a certain medium or substance such as clothing, plastics, and drywalls. Furthermore, it has a millimeter-rage of accuracy regardless of the environmental conditions such as snow, fog, rain, and dust. The frequency-modulated continuous waveform (FMCW) radar is a technology that obtains distance information from the radar by frequency modulation of continuous signals [24], but in the past, its use was limited to certain specialized applications, such as radar altimeters. However, due to the development of embedded system technology in recent years, the industry now has new applications for FMCW technology. First, the most common advantage of FMCW is that it is easy to integrate with various solid-state transmitters. Secondly, it can use the embedded system that supports real-time FFT processing to digitally calculate the distance measurement from solid-state transmitters.

On the other hand, intelligence sensors have recently combined cameras and the mmWave radar within the DNN-LSTM network for tracking moving objects [25]. LSTM is a special RNN that improves the problem of gradient disappearance and gradient explosion during the long-term training of RNN. It adds forgetting, updating, and output steps in neurons, greatly improving the performance of long-term memory. In [26] mmWave, the radar combined camera is mentioned. Furthermore, to study the position and velocity all together, the LSTM network is applied to the fall-detection method, which is based on the mmWave radar signal.

**Figure 1:**
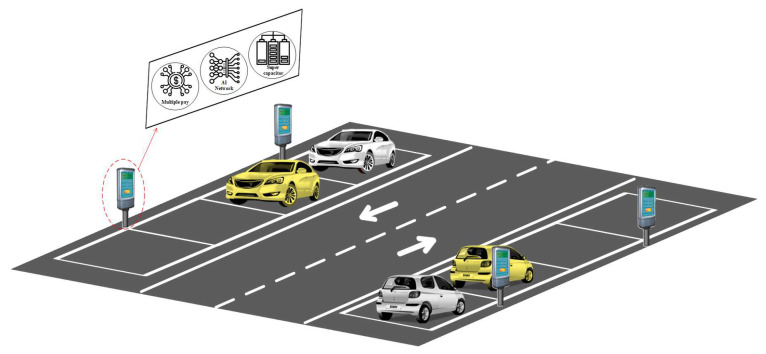
The overall concept of artificial intelligence unmanned parking meter system.

In this paper, in order to overcome a single-input image or radar heatmap input suffering from various weather conditions such as fog, rain, or twilight, we proposed a heterogeneous fusion algorithm to combine the mmWave radar heatmap image and camera image for artificial intelligence unmanned parking meter system using convolutional neural networks. The overall concept is shown in Figure 1. We choose AlexNet and MobileNet convolutional neural networks as the main architectures, mainly because the AlexNet convolutional neural network is a classic small and representative convolutional neural network model. The AlexNet network model is widely used in different fields such as medicine, agricultural disasters, and machine tool processing [27,28]. The MobileNet network uses Depthwise Separable Convolution instead of traditional convolution, which effectively reduces the number of convolution kernels without affecting the accuracy that should be achieved. In the end, each roadside unmanned parking toll node will be equipped with a set of Edge AI processing platforms, NVIDIA Jetson Nano. It is an Edge AI processor, and through embedded systems built-in acceleration technology it is used to calculate programs such as deep learning, real-time image information, system management, etc., and then the analysis results are uploaded to the management system via WiFi or 4G/5G IoT technology.

Furthermore, this research paper is organized as follows. Section 2 begins with a briefing of the Active Radar mmWave sensor module. Section 3 described the structure of convolutional neural network. The proposed heterogeneous fusion algorithm used to combine mmWave radar heatmap image and camera image will be described in Section 4. The experimental results are shown in Section 5. Section 6 concludes this paper.

## 2. mmWave Radar Technology

Table 1 lists the specifications of various millimeter-wave radar modules. From the specification Table, it can be established that in terms of range, Texas Instrument IWR6843 provides the largest detection range, which is four times that of other FMCW-based modules. Moreover, it operates at a 60 GHz high-frequency bandwidth, which is much higher than other modules. The most important feature is IWR6843, which uses the latest single-chip AOP packaging technology to integrate the radar and DSP processor, which can greatly reduce costs and reduce the module size. The proposed dual-input heterogeneous architecture can be integrated into the parking meter embedded system. Importantly, the single mmwave radar sensor can cover left and right parking lots with a single mmwave radar function to generate a heatmap for fusion. In addition, in the comparison of the field of view, the technology of IWR6843 also provides the widest field of view (azimuth and elevation), up to 130 degrees, that is, a single intelligent roadside unmanned parking toll system can cover detect two parking spaces, which can also reduce the system cost.

Therefore, the IWR6843AOP for FMCW continuous wave technology is selected for the smart roadside unmanned parking toll system in this paper. The mmWave antenna module and DSP processor are packaged directly together with AOP technology to provide 60 GHz detection bandwidth.

**Figure 2:**
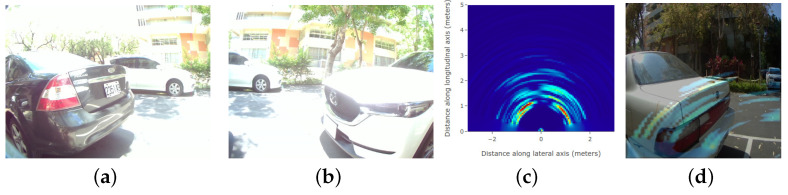
The fusion results of camera image and mmWave millimeter wave radar for parking meter system. (**a**) Left camera. (**b**) Right camera. (**c**) mmWaveR HeatMap. (**d**) mmWave Fusion.

In the parking meter system, once the single-point LiDAR receives the parking of the vehicle, firstly, the system will activate the left and right cameras to take pictures and then active the mmWave radar module followed by transforming reflection signals into heatmap information. As shown in Figure 2, from Figure 2a,b, there are two vehicles parking at the left side and right side of the parking meter system. The phase radar heat map is at a distance of 1.5 m, where Figure 2c shows the heatmap results of both left and right parking lots; there is an obvious reflection of objects, indicating that if there is a car parked on the heat map. In the end, after the fusion algorithm, Figure 2d shows the heterogeneous fusion results for neural network training.

## 3. CNN Network Comparisons

**Figure 3:**
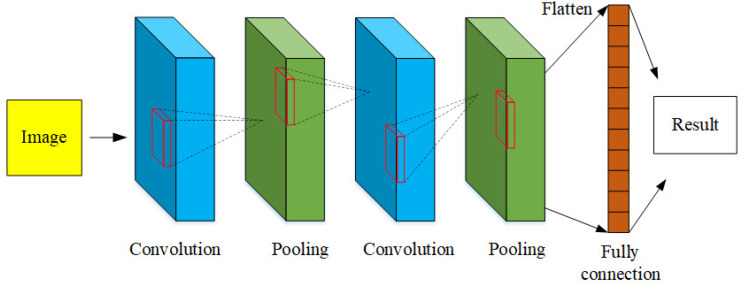
Structure of a standard convolutional neural network.

In this section, we will discuss the selection of the training network for the proposed heterogeneous fusion algorithm to combine the mmWave radar heatmap image and camera image for the artificial intelligence unmanned parking meter system. CNN is an extremely important topic in deep learning. However, a major feature of this type of neural network is convolution. Convolution is a mathematical operation. Perform feature extraction, and the extracted features will be sent to the next convolutional layer for feature extraction. This method strengthens the learning efficiency of the neural network. Among them, the network architecture includes convolution operations, pool operations, and fully connected operations. A standard convolutional neural network architecture where red rectangle frame present as the matrix convolutional is shown in Figure 3.

For the proposed heterogeneous fusion algorithm on the artificial intelligence unmanned parking meter system, we have compared AlexNet, VGG, GoogLeNet, ResNet, and MobileNet in terms of structure and complexity, as shown in Table 2, which illustrates the comparison results of different models. The reason the AlexNet is selected is because it is the foundation of all CNN networks, it is medium-sized, and it is predictable; thus, it can provide a fair point of view for the following training/inference results. Second, we choose the MobileNet because of its lightweight design is a particular need for the embedded platform of Jetson Nano. Since the experiments of unmanned parking meter systems in the project are powered by battery, low power and lightweight computing are required for MobileNet.

## 4. The Proposed Heterogeneous Fusion Algorithm

**Figure 4:**
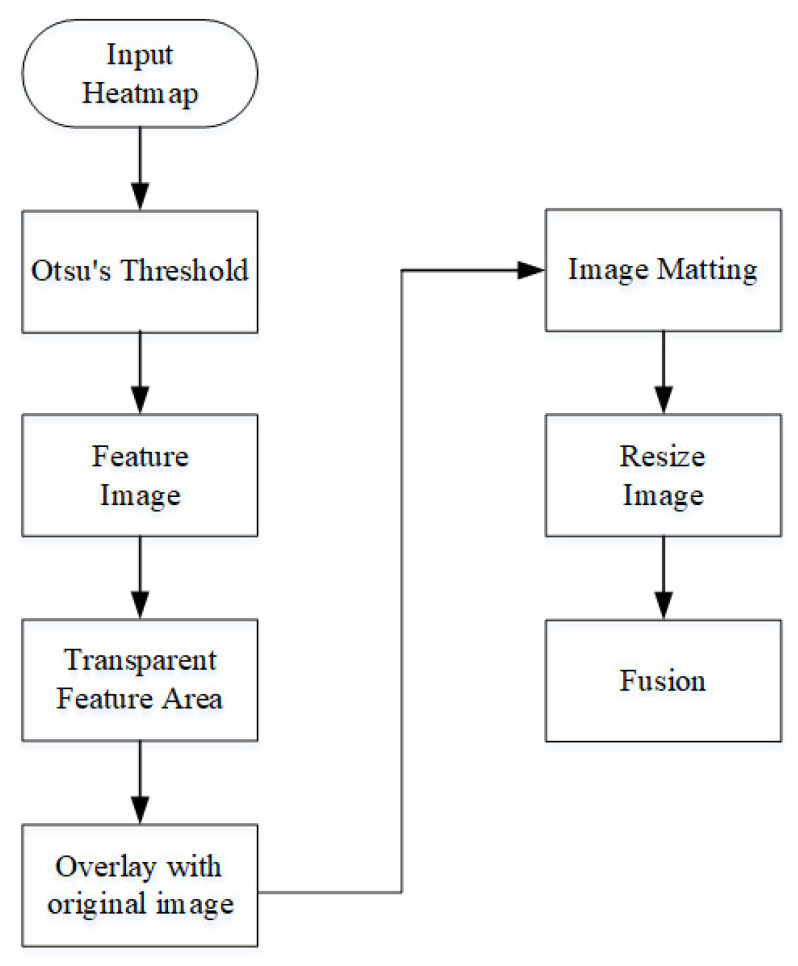
Heterogeneous fusion algorithm flowchart.

The flow chart of the proposed heterogeneous image fusion is shown in Figure 4. First, input the radar heatmap, and afterward apply Otsu binarization [32] to the radar heatmap to find the best threshold and output the black and white feature map. After that, the feature map area is transparentized, overlapped with the original radar heat map, and removed to obtain the feature area of the radar heat map. Then, adjust the image size, and finally merge with the camera image to obtain the final heterogeneous image.

**Figure 5:**
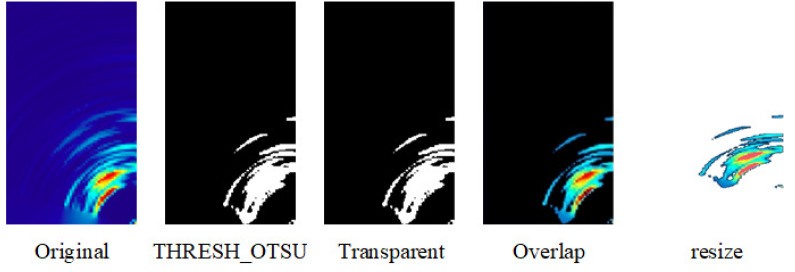
Binarization process of the mmWave radar heatmap.

The Otsu binarization method is used because it can automatically calculate the best threshold value without manually finding the matching threshold value. During the experiment, it was found that some masked radar heatmaps would be misjudged by applying the Otsu binarization method. For example, when the radar signal was very weak, the masked areas would be mistaken for characteristic areas; thus, the original radar heatmap was used instead. Carry out the Otsu binarization method to find out the characteristic area. Figure 5 shows the binarization process of the radar heatmap. From left to right, the original image, the output image using the Otsu binarization method, the feature transparency, the overlap, and the output of a complete heterogeneous image are output.

**Figure 6:**
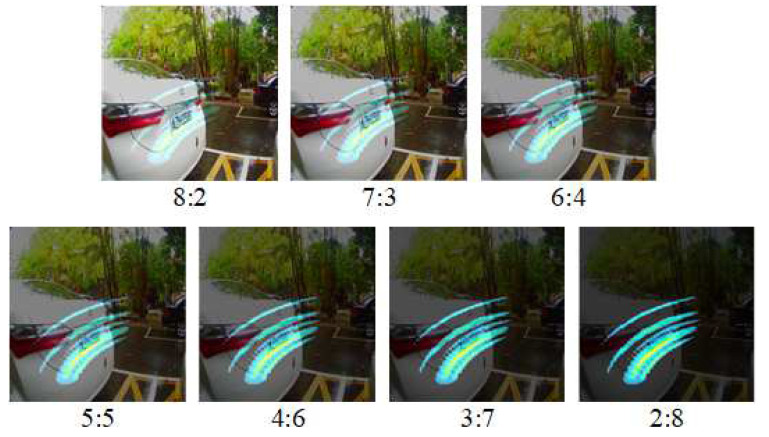
Heterogeneous image fusion with seven ratios.

Subsequently, the radar hotspot feature map and the camera image are subjected to heterogeneous image fusion. Use the cv2.addWeighted function in OpenCV to perform heterogeneous image fusion. This function can assign different weights to two images, including 2:8, 3:7, 4:6, 5:5, 6:4, 7:3, and 8:2 (the camera image ratio radar hotspot feature map), a total of seven ratios. The overall heterogeneous image fusion must be completed in four categories: left and right cars and left and right without cars. As shown in Figure 6, heterogeneous image fusions. There is a car on the left and no car on the left.

## 5. Experimental Results

### 5.1. Parking Meter System

**Figure 7:**
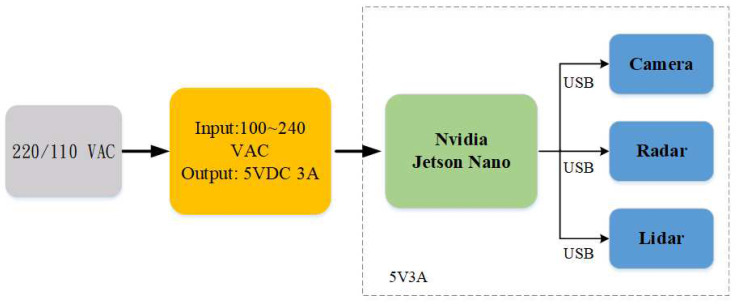
Parking meter data collector on NVIDIA Jetson Nano.

In the aspect of data collection, in order to realize the intelligent sensing machine for unmanned roadside parking timing, the data aspect must simulate the real way. This experiment uses the NVIDIA Jetson Nano embedded platform. Due to the small size of the Jetson Nano, it can be placed in an unmanned roadside parking timer smart sensing machine in a limited space. The energy consumption of the Jetson Nano is low, and there is a need to connect to the camera and mmWave radar, LiDAR, so it must use 5 volts and 3 amps of electricity to run whole system. Figure 7 shows the overall power supply terminal and the sensor setup. In terms of power supply, Jetson Nano need a power supply to convert AC 220/110 volts into 5 volts and 3 amps.

**Figure 8:**
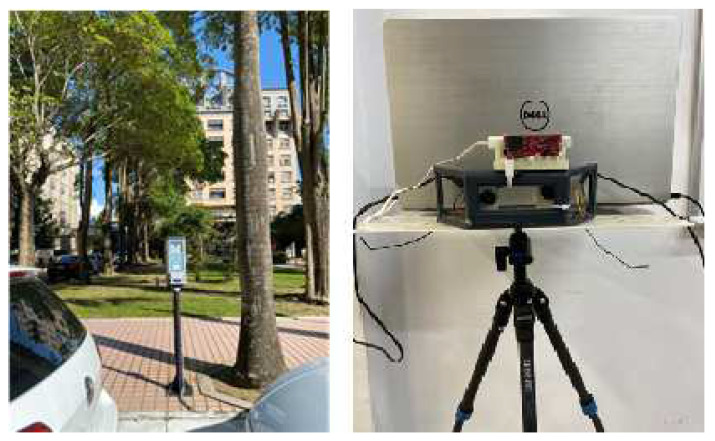
Parking meter data collector.

As shown in Figure 8, the setup of platform is to build for a radar data collection. The overall data chain can be constructed more easily and closer. The operation involves actual unmanned roadside parking timing intelligent sensing.

**Figure 9:**
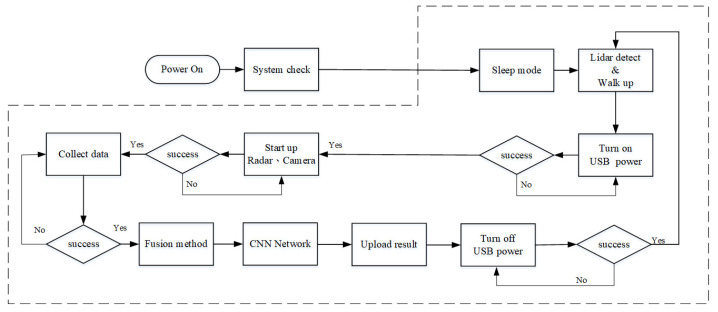
Parking meter system flow chart.

Figure 9 shows the parking meter system flow chart. It has a task-scheduling system in the Linux embedded system that can automatically collect data and quickly expand the database. The mmWave radar will first heat up after a few seconds operation, through the Uhubctl protocol, to control each USB power IO to turned on/off camera and mmWave radar. This can effectively solve the problem of the heating of the mmWave radar. Once the Lidar module detects the activities of the parking compartment, the system will turn on the camera and the mmWave radar to capture the inputs, through the proposed fusion method to detect the parking events; after few seconds, if there are no activities, the system will turn off the the camera and the mmWave radar will then return back to sleep mode.

### 5.2. Data Augmentation

**Figure 10:**
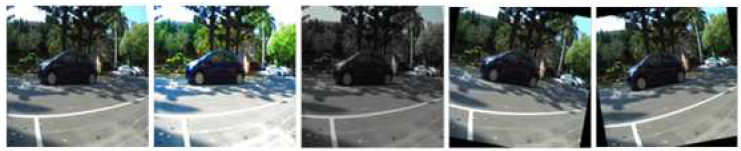
Data augmentations with original image, increasing of the brightness and darkness, rotating left 10 degrees, and rotating right 10 degrees.

In this experiment, the overall training database is 850 sets, and verification is 150 sets; later, these datasets are increased five-fold using the data-augmentation method; these include the original image, increasing the brightness and darkness of the image, and rotating the image 10 degrees to the right and 10 degrees to the left. Figure 10 shows that the camera image augmentation results, from left to right, are original image, increased brightness, decreased brightness, right rotation 10 degrees, and left rotation 10 degrees. Hence, the data augmentation training database is 4250 sets, and verification is 750 sets.

**Figure 11:**
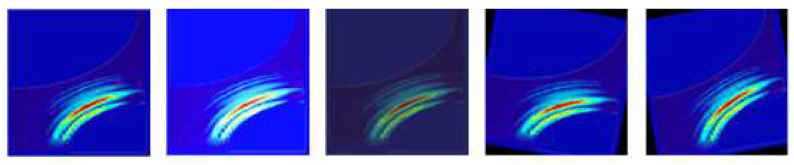
Data augmentations with original mmWave heatmap, increasing the brightness and darkness, rotating left 10 degrees, and rotating right 10 degrees.

The mmWave radar heatmap must be modified before classification and increment. Since some noise will be generated during the output of the heatmap, the noise must be masked, such as the gutter cover, etc., without affecting the judgment of the convolutional neural network model, and then the image increment will be performed. Figure 11 shows that the data augmentations of the radar heatmap, from left to right, are original image, increased brightness, decreased brightness, right rotation 10 degrees, and left rotation 10 degrees.

For comparison, initially, all the image scenes were manually annotated with non-car parking and car-parking regions with ground truth. The analysis of the results of the proposed algorithm is evaluating the number of features such as TruePositive, FalsePositive, TrueNegative, and FalseNegative values. Besides evaluating the well-known accuracy in Equation (1), the simulation environments were

- Windows 10 64-bit
- Intel Core i5-7500 3.6 GHz and DDR IV 32 GB
- Nvidia GTX 1080ti 11 GB
- CUDA Version 11
- TensorFlow Version 2.4.1
- OpenCV 4.5.1

(1) $$\begin{array}{ccc} Accuracy & = & \frac{TruePositive+TrueNegative}{TotalPopulation} \end{array}$$

### 5.3. Comparison Results

**Figure 12:**
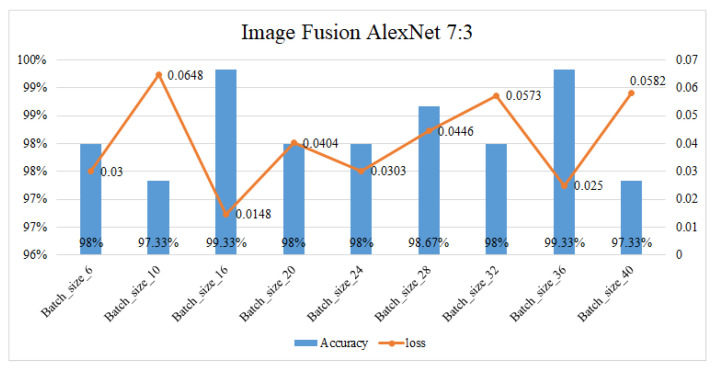
Accuracy and loss ratio when the fusion ration is 7:3 in AlexNet.

**Figure 13:**
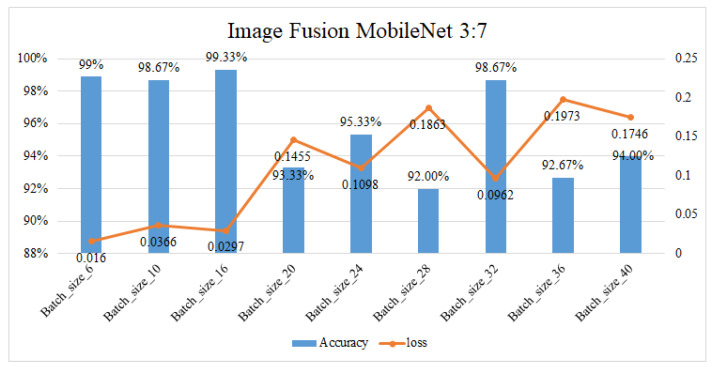
Accuracy and loss ratio when the fusion ration is 3:7 in MobileNet.

**Figure 14:**
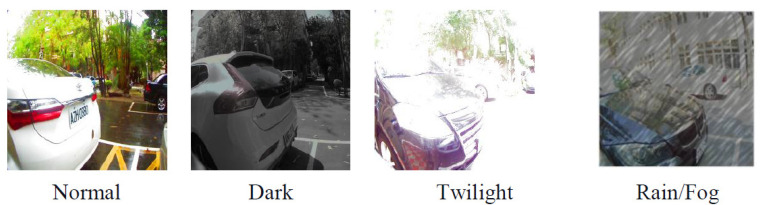
Bad condition tests on rain/fog, twilight, and dark.

**Figure 15:**
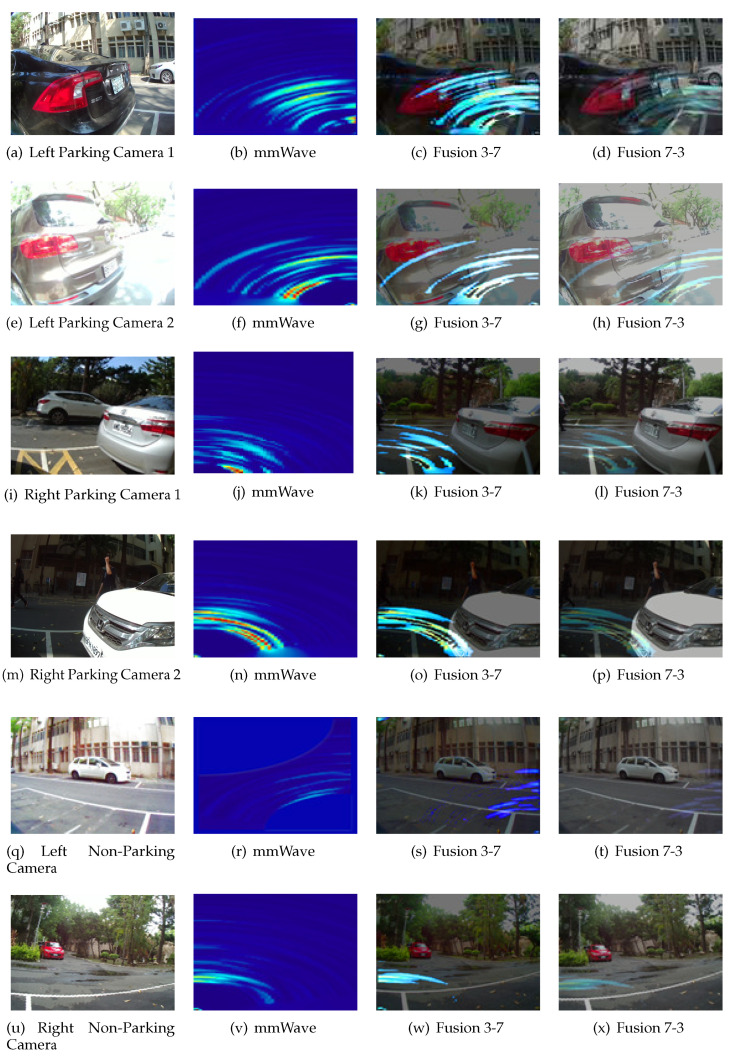
The results of the proposed algorithm to collected dataset on the street.

The AlexNet neural network and MobileNet neural network are trained with batch sizes set to 6, 10, 16, 20, 24, 28, 32, 36, and 40 to find find the best accuracy. The optimizer is set to stochastic gradient descent, loss is set to categorical crossentropy, and training will stop when the accuracy reaches 100% or a loss ratio less then 0.03. The single image results in an accuracy without the proposed fusion method that is about 96% in MobileNet and 97% in AlexNet, with both the architecture AlexNet and the MobileNet at a batch size of 16. Under the same circumstances, after applying the fusion method the single mmWave radar heatmap accuracy is about 95.83% and 86.67% with the AlexNet and the MobileNet, respectively, at a batch size of 16, representing the best results. Table 3 shows the accuracy of the proposed fusion method in two different neural network architectures. The best results of fusion ratio and training results in AlexNet are 7:3 within 99.33%, a loss ration is 0.0148 as shown in Figure 12. The best results of the fusion ratio and training results in MobileNet are 3:7 within 99.33%, and the loss ratio is 0.0297 as shown in Figure 13. In addition to normal parking conditions, we used the AlexNet fusion network to test different rations in rain, fog, dark, and twilight bad weather conditions, as shown in Table 4; once we detect whether the camera channel is too dark or light, the proposed system can switch the ratio in order to retain the best accuracy in parking lot detection, where Figure 14 shows some bad conditions. Figure 15 shows six different scenarios with left parking, right parking, non-parking, etc. It should be noticed that the sensor’s angle and position are focused on the best results of a car license plat instead of the whole parking lot because after uploading to the cloud we need this system to analyze the car license number.

## 6. Conclusions

To overcome single-input image and optimized network architecture, a heterogeneous fusion algorithm to combine the mmWave radar heatmap image and camera image for the artificial intelligence unmanned parking meter system is proposed. To avoid using the trial and error method to determine the architecture parameters of the convolutional neural network, we applied the AlexNet and MobileNet convolutional neural networks as the main architecture. In the end, each roadside unmanned parking toll node will be equipped with a set of Edge AI processing platforms, NVIDIA Jetson Nano. Judging by the experimental results, it is clear that the accuracy of the heterogeneous fusion method can reach up to 99.33% on average. It is noteworthy that the proposed fast algorithm is ideal for those low-power embedded devices that need to solve the complex road environment problem.

## Figures and Tables

**Table 1:** Specifications of radar modules.

Module	SENSE2GOL [29]	OPS241-B [30]	IWR6843 [31]
Manufacture	Infineon	OmniPreSense	Texas Instrument
Method	Doppler	FMCW	FMCW
Range	25 m	30 m	120 m
Horizontal	29${}^{\circ }$	78${}^{\circ }$	130${}^{\circ }$
Elevation	809${}^{\circ }$	N/A	130${}^{\circ }$
Frequency	24 GHz	24 GHz	60 GHz
Muddle Size	45 × 36 mm	53 × 59 mm	39 × 16 mm
Price	160 usd	170 usd	120 usd

**Table 2:** The comparsion of different CNN models.

Model	Layer	Parameters	Feature
AlexNet [17]	8 Layers	74,294,020	Dropout, ReLU.
VGGNet [19]	16/19 Layers	138,357,544	VGG16 and VGG19, Deep Network.
GoogLeNet [20]	22 Layers	6,258,500	Inception Module, Improve network resources.
ResNet50 [21]	50 Layers	25,636,712	Bottleneck Block, Identity mapping.
MobileNet [23]	28 Layers	4,253,864	Depthwise Separable Convolution, Reduction of Parameters.

**Table 3:** The weigh of fusion ratio and training results in AlexNet and MobileNet.

	AlexNet		MobileNet	
Weight	Accuracy	Loss	Accuracy	Loss
2:8	98.67%	0.0434	97.33%	0.0592
3:7	98.67%	0.0200	99.33%	0.0297
4:6	96.00%	0.0774	98.00%	0.0580
5:5	99.33%	0.0277	98.67%	0.0326
6:4	98.00%	0.0448	99.33%	0.0348
7:3	99.33%	0.0148	97.33%	0.0854
8:2	96.67%	0.1332	97.33%	0.0799

**Table 4:** The rain, fog, dark, and twilight bad weather conditions in Alexnet fusion ratio.

	AlexNet			
Weight	Normal	Twilight	Dark	Rain
2:8	98.67%	93.75%	96.25%	99.00%
3:7	98.67%	90.00%	93.75%	100.0%
4:6	96.00%	92.50%	92.50%	98.00%
5:5	99.33%	96.25%	91.25%	94.00%
6:4	98.00%	96.25%	88.75%	94.00%
7:3	99.33%	93.75%	95.00%	95.00%
8:2	96.67%	87.50%	95.00%	87.00%

## Data Availability

Not applicable.
